# CT Characteristics of Osteolathyrism in a Pig Model of β‐Aminopropionitrile and Surgery‐Induced Aortic Aneurysm

**DOI:** 10.1111/vru.70057

**Published:** 2025-06-22

**Authors:** Jeryl Jones, Matt Breed, Guillermo Rimoldi, Naren Vyavahare, Ahmed Ali

**Affiliations:** ^1^ Department of Animal and Veterinary Sciences; College of Agriculture, Forestry, and Life Sciences; Clemson University Clemson South Carolina USA; ^2^ Office of Animal Resources Clemson South Carolina USA; ^3^ Veterinary Diagnostic Center Clemson Livestock Poultry Health, Clemson University Clemson South Carolina USA; ^4^ Department of Bioengineering Clemson University Clemson South Carolina USA

**Keywords:** bone proliferation, lathyrism, porcine, swine

## Abstract

Pigs are established animal models for translational research studies of aortic aneurysms in humans. A recent publication recommended adding oral treatment of β‐aminopropionitrile (BAPN) as a technique for enhancing surgically induced aortic aneurysm formation in pig models. The aims of the current prospective pilot study were to build on previous studies and develop a reproducible protocol for future use in our group's longitudinal research studies. Two, 3‐month‐old, male castrated, Yorkshire pigs were included. Pigs were treated with oral BAPN, and aortic aneurysms were induced using a surgical protocol we had developed and refined in previous pilot studies. Pigs were evaluated using whole‐body CT and full necropsy examinations at the study's 4‐week termination timepoint. We identified and described previously unreported CT characteristics of osteolathyrism as an unexpected adverse event in both pigs. Authors recommend using lower doses of BAPN and monitoring pigs closely for bone changes in future studies.

## 1 Introduction

Pigs are established animal models for researchers who study mechanisms of, and novel treatments for, aortic aneurysms in humans [1, 2, 3, 4]. Cullen et al. [4] reported pathologic evidence of increased aortic dilation in research pigs with surgically induced abdominal aortic aneurysms that were also treated daily with β‐aminopropionitrile (BAPN) at a dosage of 0.15 g/kg. The objectives of the current study were to build upon this prior research and develop a reproducible protocol for use in our group's longitudinal research studies.

## 2 Materials and Methods

### 2.1 Animal Selection Criteria

The study was a prospective, pilot design and part of a larger translational research study on novel treatments for aortic aneurysms. All procedures were approved by and conducted in accordance with the Clemson University Institutional Animal Care and Use Committee (AUP 2023‐0097). Two, 3‐month‐old, male castrated, 15–20 kg, Yorkshire pigs were acquired from a research vendor and transported to an AAALAC‐accredited animal research center (Godley‐Snell Research Center, Clemson University). An ACLAM‐certified attending veterinarian (MB) examined the pigs and determined them to be clinically normal. Pigs were housed, acclimated, and managed by animal research staff using their standard operating procedures. Pigs were fed a commercially available pig diet (Mazuri Exotic Animal Nutrition, PO Box 66812, St. Louis, MO 63166).

*Beta*‐aminopropionitrile (BAPN) was administered orally, mixed in a treat, once daily beginning 1 week before surgery and continued for 4 weeks after surgery (0.15 g/kg, 3‐aminopropionitrite fumarate, [fumarate salt of BAPN], ThermoFisher Scientific) [4] Pigs were anesthetized as per the research center's standard operating procedures and prepared for aseptic surgery. Animals were premedicated with atropine and acepromazine. Anesthesia was induced with ketamine, the animals were intubated and maintained under anesthesia with isoflurane. Analgesia was provided locally at the incision with bupivacaine, and systemically with buprenorphine and ketoprofen. Preoperative antibiotics were administered (Tulathromycin, Draxxin, 2.5 mg/kg, IM, 100 mg/mL, Zoetis). The attending veterinarian (MB) and a veterinarian with 17 years of surgery experience (AA) induced aortic aneurysm formation for both pigs using a surgical protocol adapted from previously published studies [4, 5] and refined in two of our previous pilot studies. An incision approximately 15 cm long in the abdomen was made parallel to the prepuce. After entering the abdomen, the intestines were kept moist with sterile saline‐soaked laparotomy sponges and gently reflected to locate the abdominal aorta. Approximately 5 cm of the descending abdominal aorta was exposed between the renal arteries and the aortic bifurcation. Up to 5 mL of *Clostridium histolyticum* collagenase/porcine pancreatic elastase cocktail solution was injected into a 5 cm segment of the ventral wall of the abdominal aorta using a hand‐bent needle (26‐gauge). Each injection point was injected with up to 0.4 mL of enzyme cocktail, and injections were spaced approximately 0.5 cm apart. The abdomen was then closed in three layers with absorbable sutures. Post‐operative analgesia was provided with buprenorphine (Simbadol, 0.05 mg/kg, IM, 1.8 mg/mL, Zoetis) and ketoprofen (Ketofen, 2.0 mg/kg, IM, 100 mg/mL, Zoetis). Pigs were monitored and managed by the attending veterinarian and research center staff for the remainder of the study.

### 2.2 Data Recording and Analysis

#### 2.2.1 Computed Tomography

At the 4‐week termination timepoint for the study, whole‐body CT scans were acquired by a research center technician, using a 16‐slice CT scanner (Toshiba Aquilion TSX‐101A, GE Healthcare, Chicago, IL) and a standardized image acquisition protocol developed by an ACVR‐certified veterinary radiologist (J. J.). One pig was anesthetized as previously described for CT scanning. Immediately following image collection, the pig was humanely euthanized with a pentobarbital overdose without anesthesia recovery. One pig was imaged post‐mortem. Both pigs were positioned in dorsal recumbency with the forelimbs extended cranially and the hindlimbs extended caudally. Stifles were positioned in adduction and maintained using gauze ties. A scan of the thoracic region was acquired from the level of the last cervical vertebra to the caudal margins of the ribs (helical mode, 3 mm slice thickness, 25 kg abdomen protocol, acquired with standard body algorithm and reconstructed with bone algorithm). A scan of the abdomen was also acquired from the cranial margin of the diaphragm to the caudal margin of the ischium using the same technical parameters. The veterinary radiologist interpreted images using a dedicated image analysis workstation (Mac OS High Sierra, 10.13.6, MacPro Quad Core, Apple, Inc. Cupertino, CA; Horos https://horosproject.org/).

#### 2.2.2 Pathology

Immediately after CT scanning, the bodies of the pigs were placed in a cooler and transported to a veterinary diagnostic center (Clemson Livestock Poultry Health Center, Clemson University). An ACVP‐certified veterinary pathologist (G R) performed full necropsy examinations.

## 3 Results

### 3.1 Computed Tomographic Findings

**FIGURE 1:**
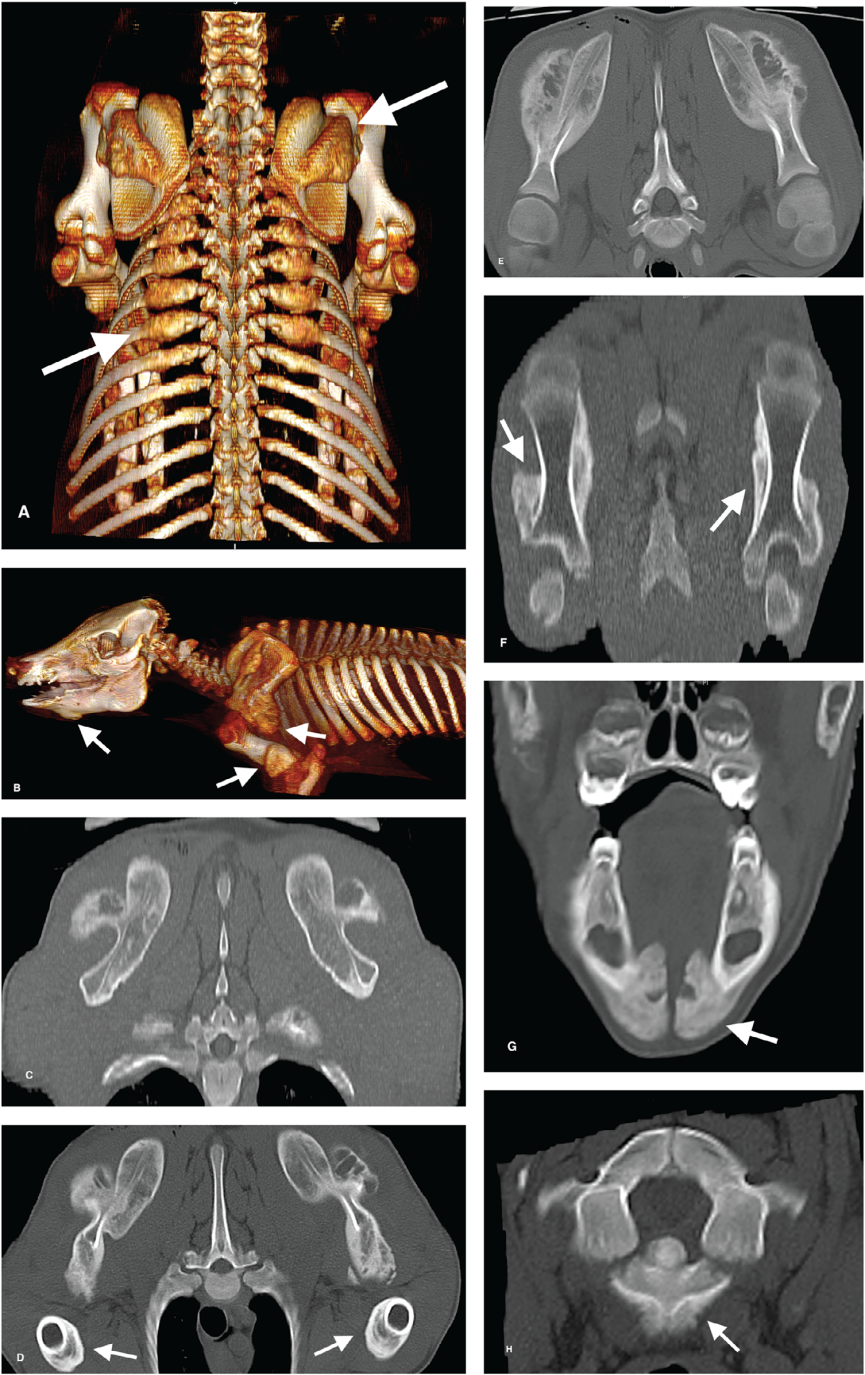
CT images illustrating bilaterally symmetrical shape distortions and chronic/active periosteal reactions with single layer, multilayer, solid, and disorganized characteristics involving multiple bones of the appendicular and axial skeleton in the cranial portion of the body for both pigs (bone window display settings, see Methods section for technical parameters). Arrows in each of the images indicate locations with bone shape distortions and periosteal reactions. A, Three‐dimensional (3D) volume‐rendered (VR) image of pig 2, dorsal view. B, 3D VR image of pig 1, left lateral view. C, 3D multiplanar reformatted (MPR) transverse image of the scapulae in pig 2. D, 3D MPR transverse image of the scapulae and humeri in pig 2. E, 3D MPR dorsal planar image of scapulae in pig 2. F, 3D MPR dorsal planar image of both humeri for pig 1. G, 3D MPR transverse image of mandibles for pig 1. H, 3D MPR transverse image of the C1‐2 vertebrae for pig 1.

**FIGURE 2:**
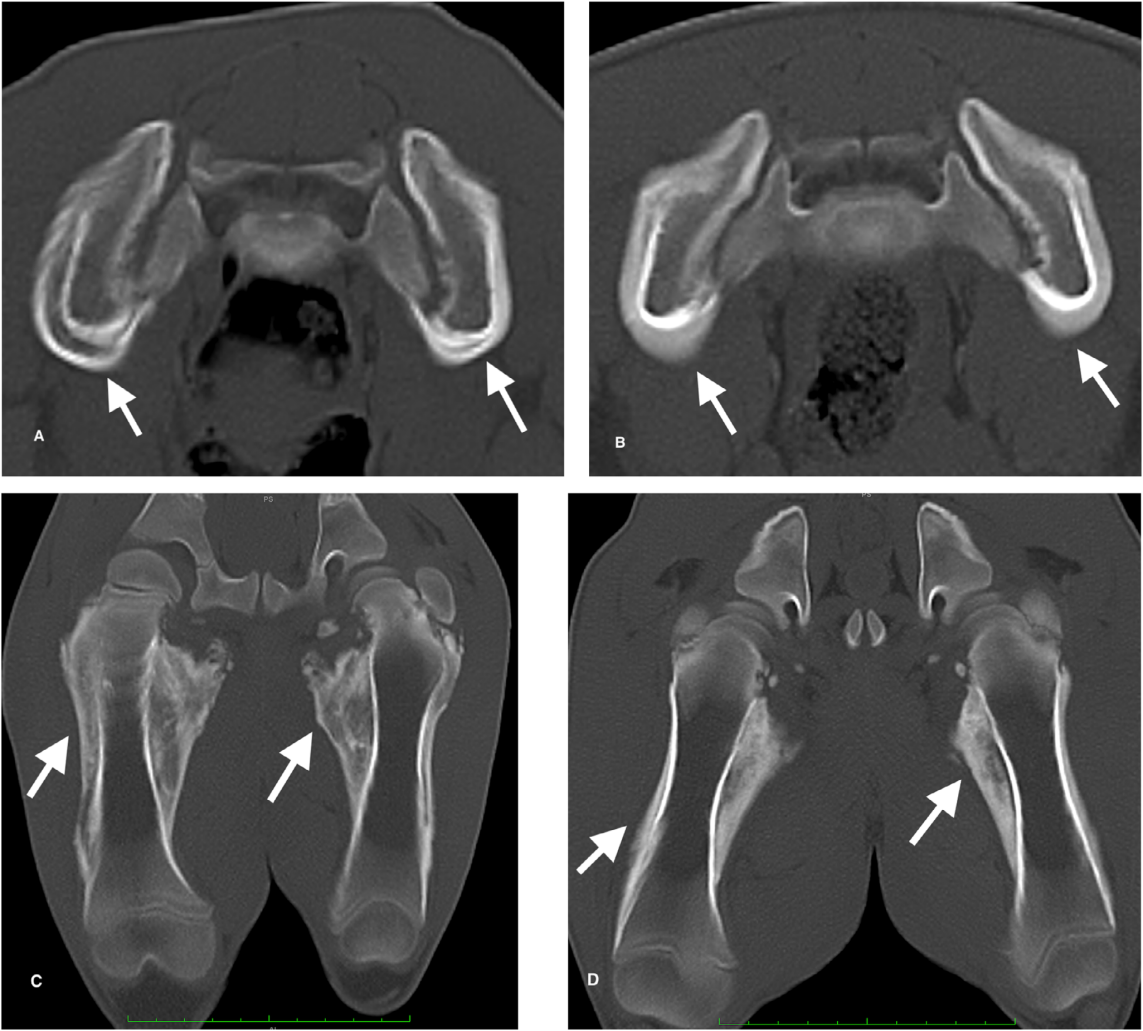
CT images documenting similar periosteal reactions in ilia and femurs for both pigs (bone window display settings, see Methods section for technical parameters). Arrows in each of the images indicate locations with periosteal reactions. A, 3D MPR transverse image of ilia for pig 1. B, 3D MPR transverse image of ilia for pig 2. C, 3D MPR dorsal planar image of femurs for pig 1. D, 3D MPR dorsal planar image of femurs for pig 2.

Bilaterally symmetrical shape distortions and chronic/active periosteal reactions were present in multiple bones of the axial and appendicular skeleton for both pigs (Figures 1 and 2). Affected bones of the axial skeleton included the mandibles, cervicothoracic vertebrae, ribs, and ilia. Affected bones of the appendicular skeleton included the scapulae, humeri, and femurs. Periosteal reactions exhibited single‐layer, multilayer, solid, and disorganized characteristics. Findings were more severe in the cranial portion of the body. Long bone findings were predominantly diaphyseal. Primary differential diagnoses included metabolic or nutritional disease.

#### 3.1.1 Pathologic Findings

**FIGURE 3:**
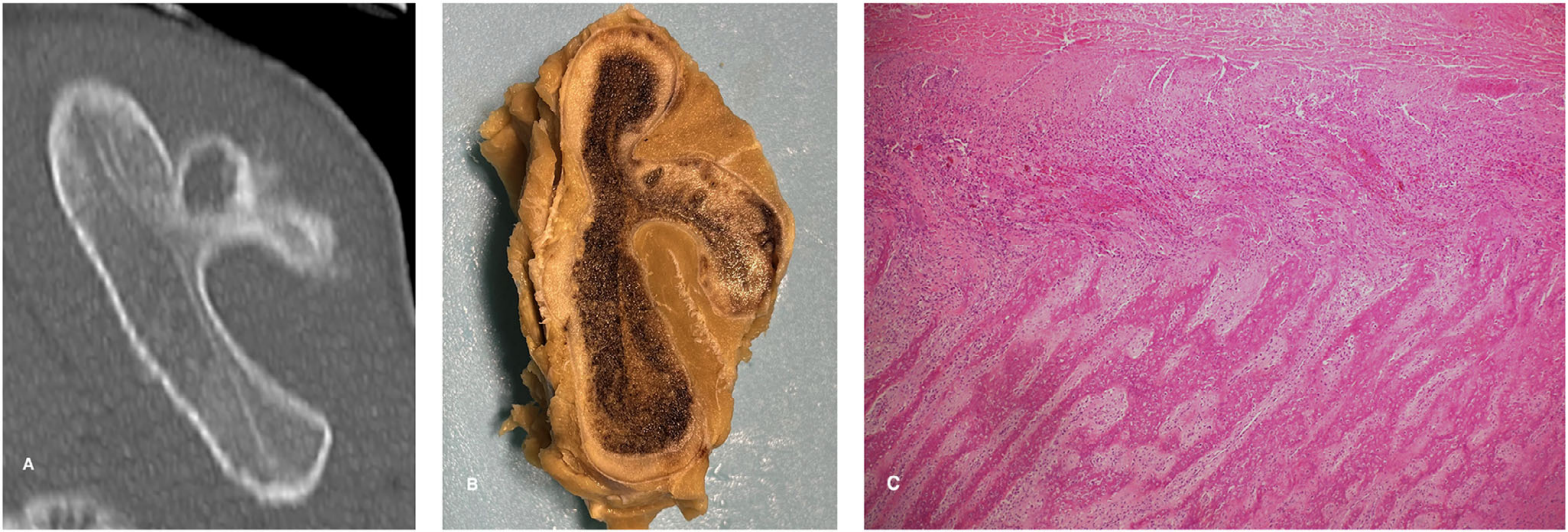
Comparison of CT and pathologic findings in the left scapula for pig 2. A, 3D MPR, zoomed transverse image of the left scapula illustrating shape distortion and chronic/active periosteal reactions. B, Transverse cross‐section illustrating a severely thickened scapular spine with rounded edges and curved shape (formalin‐fixed sample). C, Histologic image illustrating periosteum markedly expanded by the proliferation of poorly arranged spindle cells, forming short streaming and interlacing bundles. Subjacent to the expanded periosteum proliferation of immature and mature bone, composed of poorly organized, haphazardly arranged, variably sized, spicules of osteoid bone spicules. The proliferations typically do not incorporate hematopoietic elements within marrow spaces and are filled with poorly cellular connective tissue (H/E 40X).

The main changes detected during necropsy on the bones of both animals were in the scapulae, and these bones were particularly thoroughly examined (Figure 3). They were found to be bilaterally and symmetrically deformed, markedly thickened with rounded edges and curved spines on transverse sections. Multiple sections were obtained for histological examination. Decalcification of bones was performed with a formic acid‐containing commercial solution (Cal‐Ex II Fixative Decalcifier, Thermo Fisher Scientific, Waltham, MA). The decalcification process took less than a week when it was expected to require at least 4 weeks. Histology sections were obtained after the routine paraffin‐embedding process to obtain 5 µm‐thick sections that were stained with hematoxylin and eosin (H&E). Multiple histological changes were detected on the scapulae, the main ones affecting primarily the crests and borders. In these areas, the periosteum was markedly expanded by the proliferation of poorly arranged spindle cells, forming short streaming and interlacing bundles. Subjacent to the expanded periosteum, there was a proliferation of immature and mature bone, composed of poorly organized, haphazardly arranged, variably sized spicules of osteoid bone. These proliferations typically did not incorporate hematopoietic elements within marrow spaces and were filled with poorly cellular connective tissue. The changes were summarized as an atypical periosteal fibroplasia, with osseous differentiation (exostoses) and myelofibrosis consistent with osteolathyrism [6].

## 4 Discussion

The intent of the current pilot study was to provide background for our group's larger translational research study on novel interventions for aortic aneurysms using swine models. We partially replicated protocols from a 2019 publication describing the use of BAPN at a dose of 0.15 g/kg as a technique for enhancing surgically induced aortic aneurysm formation in pig models [4]. We evaluated our pigs using whole‐body CT at the 4‐week terminal timepoint of the study and identified severe deformations and chronic/active periosteal reactions involving multiple bones of the appendicular and axial skeleton. Pathologic examinations confirmed a diagnosis of osteolathyrism. This adverse event was unexpected because the previous publication did not report any bone changes at the 4‐week terminal timepoint for their pigs.

We contacted the authors of the previous study to ask if they had observed any bone changes in their pigs and received a response that they were “no longer using swine models for various reasons”. To rule out nutritional disease as another possible cause for the bone changes seen in our pigs, we retrieved CT images of pigs from one of our previous pilot studies. Pigs in that study were fed the same commercial pig diet and underwent the same surgical procedures but were not treated with BAPN. Skeletal structures in those pigs appeared normal. We conducted an additional literature search and found that there were no further publications describing BAPN treatments in pigs since 2019. One 1969 article was found describing pathologic characteristics of osteolathyrism in pigs treated with BAPN as part of another experimental study [7]. Pathologic findings illustrated in this previous report were comparable to the CT and pathologic findings observed in our pigs. The BAPN dosages in the previous paper varied from 0.2 to 3 g/kg/day, comparable to the 0.15 g/kg daily dosage we used for the pigs in the current study. Review articles from 1957 and 1974 described BAPN as a compound that naturally occurs in plants and seeds of the genus *Lathyrus* [8, 9]. Because this compound had been reported to cause aortic aneurysms in humans who ingested large quantities of seeds from these plants, especially *Lathyrus sativus* (grass pea or chickpea), BAPN has been described as a treatment for experimentally inducing aortic aneurysms in translational research studies using animal models. However, this compound has also been reported to cause neurologic disease (neurolathyrism) in humans and a wide variety of animal species, and bone disease (osteolathyrism) in research animals. One of the proposed mechanisms for BAPN‐induced osteolathyrism in young, rapidly growing animals was described as “deposition of tropocollagen or soluble collagen in the cartilage matrix, with a resulting increase in connective tissue fragility and collagen extractability”. A 1992 article using rat models provided additional evidence that BAPN was a potent inhibitor of lysyl oxidase, an important enzyme necessary for collagen cross‐linking [10]. In addition to rats and pigs, experimentally induced osteolathyrism has also been reported in guinea pigs, cats, rabbits, chickens, hamsters, dogs, turkeys, rhesus monkeys, frogs, and newts fed BAPN or other *Lathyrus spp*. compounds [9]. No studies describing naturally occurring osteolathyrism in animals were found. No published reports were found describing CT characteristics of osteolathyrism in pigs or other species.

Polyostotic periosteal reactions and bone malformations have also been reported in humans, pigs, and other animals with rickets and fibrous osteodystrophy [11, 12, 13]. Proposed etiologies have included abnormal parathyroid hormone activity; imbalances of dietary phosphorus, calcium, or vitamin D; mineral deficiencies; toxicities; and genetic abnormalities. We ruled out etiologies other than BAPN‐induced osteolathyrism in the current pigs based on the absence of comparable CT findings in a previous pilot study using pigs that were not treated with BAPN. We also recently completed a new pilot study that included one pig administered a lower dosage of BAPN and one pig not treated with BAPN. Both pigs had the same genetics, housing, diet, and surgical interventions as the current pigs. Whole‐body CT scans were again acquired in these two new pigs, and no periosteal reactions or bone malformations were identified.

In conclusion, the current study introduced previously unreported CT characteristics of osteolathyrism as an adverse effect of administering oral BAPN at a daily dose of 0.15 g/kg in pig translational research models of aortic aneurysm. Authors therefore recommend using lower doses of BAPN and monitoring pigs closely for bone changes in future research studies.

## Conflicts of Interest

The authors declare no conflicts of interest.

## Previous Presentation or Publication Disclosure

None.

## Reporting Checklist Disclosure

The STROBE‐Vet Checklist was used.

## Data Availability

The data that support the findings of this study are available from the corresponding author upon reasonable request.
